# *SLC4A11* and *MFSD3* Gene Expression Changes in Deoxynivalenol Treated IPEC-J2 Cells

**DOI:** 10.3389/fgene.2021.697883

**Published:** 2021-07-21

**Authors:** Yafei Xu, Xiaolei Chen, Luchen Yu, Yi Wang, Haifei Wang, Zhengchang Wu, Shenglong Wu, Wenbin Bao

**Affiliations:** ^1^Key Laboratory for Animal Genetic, Breeding, Reproduction and Molecular Design of Jiangsu Province, College of Animal Science and Technology, Yangzhou University, Yangzhou, China; ^2^Joint International Research Laboratory of Agriculture and Agri-Product Safety, Yangzhou University, Yangzhou, China

**Keywords:** pig, DON, *SLC4A11*, *MFSD3*, methylation regulation

## Abstract

Deoxynivalenol (DON) caused serious cytotoxicity for animal cells. However, genes involved in regulating DON toxicity and the underlying molecular mechanisms remain largely unknown. This study explored the role of *SLC4A11* and *MFSD3* in alleviating DON toxicity and analyzed the DNA methylation changes of these two genes. Viability and cell cycle analysis showed that DON exposure decreased the IPEC-J2 viability (*P* < 0.01), blocked the cell cycle in the G2/M phase (*P* < 0.01), and increased the rate of apoptosis (*P* < 0.05). Expression of the *SLC4A11* and *MFSD3* genes was significantly downregulated upon DON exposure (*P* < 0.01). Overexpression of *SLC4A11* and *MFSD3* can enhance the cell viability (*P* < 0.01). DNA methylation assays indicated that promoter methylation of *SLC4A11* (mC-1 and mC-23) and *MFSD3* (mC-1 and mC-12) were significantly higher compared with those in the controls and correlated negatively with mRNA expression (*P* < 0.05). Further analysis showed that mC-1 of *SLC4A11* and *MFSD3* was located in transcription factor binding sites for NF-1 and Sp1. Our findings revealed the novel biological functions of porcine *SLC4A11* and *MFSD3* genes in regulating the cytotoxic effects induced by DON, and may contribute to the detection of biomarkers and drug targets for predicting and eliminating the potential toxicity of DON.

## Introduction

Deoxynivalenol (DON), belonging to the family of trichothecenes, is one of the metabolites of Fusarium, induces emesis in swine, leading to it being known as “vomitoxin” (Pestka and Smolinski, 2005; Pestka, 2010; Borutova et al., 2012). DON is extremely harmful to pigs, and its main toxic effects are antifeeding, vomiting, decreased immunity, necrosis of the digestive tract, decreased reproductive performance, and finally, death (Eriksen and Pettersson, 2004). Early research suggested that DON induced the apoptosis of piglet mesenteric lymph node cells, stimulated immune cells to secrete cytokines, and resulted in intestinal inflammation by destroying the intestinal barrier and inducing inflammation (Pestka, 2010; Gerez et al., 2015). Once the intestinal barrier is damaged, in addition to affecting the digestion and absorption of pigs, it is likely to cause other intestinal diseases, which will seriously affect the growth and development of pigs, resulting in huge economic losses to the pig industry. Therefore, it is necessary to determine the toxic effects and mechanism of DON, which will contribute to discovering new methods of prevention and treatment of diseases induced by DON.

Epigenetic modification is a type of gene regulation that is ubiquitous in all organisms, and is essential to maintain the normal life activities of mammals (Shi and Wu, 2009). Epigenetic modification includes DNA methylation, histone modification, and non-coding RNA regulation, among which DNA methylation has attracted much attention from epigenetic modification researchers (Dahl and Guldberg, 2003). DNA methylation regulates gene expression by affecting chromatin structure, DNA conformation, chromosome stability, and DNA-protein interaction. In eukaryotes, it plays an important role in cell differentiation, embryonic development, environmental adaptation, and the development of diseases (Deng, 2014; Dai et al., 2019). After DON exposure, the levels of methyltransferase H3K4me2 increased significantly on *EZH2*, encoding enhancer of Zeste 2 polycomb repressive complex 2 Subunit, a key regulator in the early development of oocytes. Thus, DON can cause the interruption of oocyte maturation by changing epigenetic modifications (Han et al., 2016). Studies have also reported increased DNA methylation levels and the expression of H3K9me3 and H4K20me3 in the oocytes of mice fed a diet containing DON, and that the expression levels of H3K27me3 and H4K20me2 were reduced, revealing that epigenetic modifications might be one of the reasons for the decrease of the level of oocyte development (Zhu et al., 2014). These studies showed that epigenetic modification plays an important regulatory role in the process of DON induction.

Our previous transcriptome analysis in IPEC-J2 cells found significant decreases in the expression levels of the *SLC4A11* (encoding solute carrier family 4 member 11) and *MFSD3* (encoding major facilitator superfamily domain containing 3) genes in response to DON (Wang et al., 2019). *SLC4A11* and *MFSD3* are both membrane-bound solute carriers (SLCs), which maintain nutrient uptake, ion transport, and waste removal associated with physiological functions (Perland et al., 2017). *SLC4A11* is an electrogenic Na/borate cotransporter that stimulates cell growth and proliferation by increasing intracellular borate levels and activating the mitogen activated protein kinase (MAPK) pathway (Jiao et al., 2007; Lopez et al., 2009). *MFSD3* is a kind of membrane-bound solute carrier that belongs to the major facilitator superfamily (MFS), which is the largest phylogenetic group of SLCs in humans (Nicoletti et al., 2019). Studies reported that the expression of *MFSD3* was associated with nutrient intake and adipose tissue homeostasis (Hoglund et al., 2011). Therefore, *SLC4A11* and *MFSD3* may play an important role in DON-induced cell damage, we further explored the expression regulation mechanism of *SLC4A11* and *MFSD3* genes associated with the activity of IPEC-J2 cells induced by DON. We examined the effects of DON on the viability, cell cycle, and apoptosis of IPEC-J2 cells, as well as the regulation of *SLC4A11* and *MFSD3* expression levels in IPEC-J2 cells induced by DON, including a comprehensive analysis of the degree of methylation and expression changes of these two genes. The present study explored the regulatory role of *SLC4A11* and *MFSD3* in resisting DON-induced cytotoxicity. Better understanding of DON pathogenesis and identification of the responsive genes provided the theoretical basis for further study of the molecular regulation mechanism of DNA methylation modification in DON-induced cytotoxicity, and may contribute to the identification of biomarkers and drug targets for DON contamination.

## Materials and Methods

### Ethics Statement

The animal study proposal was approved by the Institutional Animal Care and Use Committee (IACUC) of the Yangzhou University Animal Experiments Ethics Committee [permit number: SYXK (Su) IACUC 2012-0029]. All experimental methods were conducted in accordance with the related guidelines and regulations.

### Cell Culture

The IPEC-J2 cells were preserved in our laboratory, and cultured in Dulbecco’s modified Eagle’s medium (DMEM) containing 10% fetal bovine serum (FBS) and 1% penicillin streptomycin (1 mg/mL) at 37°C with 5% CO2.

### Cell Viability

IPEC-J2 cells were cultivated in 96-well plates at a density of 2 × 10^3^ cells/well and cultured for 24 h. Based on a previous study, (Wang et al., 2019) we could see that treatment with a DON (Sigma, Germany) concentration of 1 μg/ml for 48 h induces cytotoxicity in IPEC-J2 cells. When the cells reached 70–80% confluence, they were incubated with DON (1 μg/mL) for 24, 48, and 72 h. Cell viability was assessed using a Cell Counting Kit-8 (MedChemExpress, Monmouth Junction, NJ, United States) according to the manufacturer’s protocol. The absorbance was measured on a Tecan Infinite Pro (Sunrise, Tecan, Switzerland) at 450 nm.

### Cell Apoptosis Assay

IPEC-J2 cells were seeded into six-well plates at a density of 2 × 10^5^ cells/well and randomly assigned into a control group and a DON treated group. When the cells reached 70–80% confluence, they were incubated with DON (1 μg/mL) for 48 h in the DON treated group. Subsequently, cells were collected, and stained with Annexin V-FITC according to the instructions of the Apoptosis Detection kit (Solarbio, Beijing, China). Finally, apoptosis was analyzed using a Flow Cytometer (FAC Scan, Becton Dickinson, Franklin Lakes, NJ, United States) within 1 h.

### Cell Cycle Analysis

First, IPEC-J2 cells were cultured in a six-well plates and incubated at 37°C with 5% CO_2_ overnight and divided into a control group and a DON treated group. Then, digestion was performed with trypsin without EDTA followed by washing twice in pre-cooled phosphate-buffered saline. Cell cycle analysis was performed according to a Cell Cycle and Apoptosis Analysis Kit (Beyotime Institute of Biotechnology, Jiangsu, China) and the percentage of the cell population at a particular phase was estimated using ModFit LT for Windows V3.1 (Verity Software House, Topsham, ME, United States).

### RNA Extraction and Quantitative Real Time Reverse Transcription PCR

Total RNA was extracted from IPEC-J2 cells using Trizol. cDNA was reverse transcribed from the mRNA was reverse transcribed using a HiScript II Q RT SuperMix kit (Vazyme Biotech Co., Ltd., Nanjing, China). The cDNA was used as the template for the quantitative real-time PCR (qPCR) step using a fast real-time PCR system (ABI Step One Plus; Applied Biosystems, Foster City, CA, United States). *GAPDH* (encoding glyceraldehyde-3-phosphate dehydrogenase) was used as the control gene. The relative expression levels were determined using the 2^–ΔΔ*CT*^ method (Livak and Schmittgen, 2001) and overexpression efficiency was also calculated using the 2^–ΔΔ*CT*^ method. The primers used are shown in Table 1. All primers were synthesized by Sangon (Shanghai, China).

**TABLE 1:** The primer sequence of genes for quantitative real-time PCR (qPCR) and their sequences.

**Gene**	**Primer sequence**	**Product length (bp)**
*SLC4A11-q1*	F: AGTAGTAGGGAGCAGGGTGG	234
	R: AAGCAAGCAGAGAGTGAGCC	
*MFSD3-q2*	F: CGCCTCAGCCATCAGAACCCCGCCG	224
	R: TGAGCCCACAATGGAACAGA	
*GAPDH-q3*	F: GGTCGGAGTGAACGGATTT	245
	R: ATTTGATGTTGGCGGGAT	
*SLC4A11-c1*	F: CGAGCTCATGTCACAGAGTGGATACCC	*2631*
	R: AGCTTTGTTTAAACTCAATGATTCGGGGCAGCAG	
*MFSD3-c2*	F: CGAGCTCATGCACGGGAAGCTGCTGGT	*1635*
	R: AGCTTTGTTTAAACTCAGCCAAGGCTCCGGCCAG	
*SLC4A11-p1*	F: GTTATTTTGGGAAGGAAAAAGTT	*345*
	R: CCCTAATATCAACTTTAAATATATAACC	
*MFSD3-p2*	F: ATTCTGCCCCCCA	*260*
	R: GCCTCCAAGCAGCCAA	

*Note q1–q3 stands for qPCR primers. c1–c2 stands for overexpression primers. The underlined sequences represent restriction sites. p1–p2 stands for BSP primers, respectively.*

### Construction of Overexpression Cell Lines

Primers with *Sac*I and *Pme*I restriction enzyme sites were designed according to the coding sequences (CDS) of porcine *SLC4A11* (XM_021077564.1) and *MFSD3* (XM_021090521.1) gene published in the GenBank database^1^ (Table 1). The primers were used to amplify the *SLC4A11* and *MFSD3* genes, which were ligated into the florescence expression vector, pEGFP–C1, after double digestion. The sequences of the *SLC4A11* and *MFSD3* overexpression vectors were confirmed by Sangon sequencing. Cells were incubated with 20 nM *SLC4A11* and *MFSD3* overexpression vector or negative control using Jet PRIME (Polyplus, Illkirch, France). After transfection, stable cells lines were established by incubation with geneticin (800 μg/mL) (Invivogen, Toulouse, France) for 15 days.

### Bioinformatic Analysis and Primer Design

Analysis and identification of the promoter region, CpG islands and transcription factor binding sites in the 5′ flanking regions of *SLC4A11* and *MFSD3* were performed using the online tool MethPrimer and Alibaba (Li and Dahiya, 2002). Based on this analysis and the location of the predicted CpG islands, bisulfite-sequencing PCR (BSP) primers were designed using MethPrimer (Table 1).

### Genomic DNA Preparation and Promoter Methylation Analysis

Genomic DNA was extracted from IPEC-J2 cells using a Universal DNA purification kit (Tiangen, Beijing, China), following the manufacturer’s instructions. The genomic DNA was treated with bisulfite and used as the template for PCR (ZymoTaq PreMix; Zymo Research, Irvine, CA, United States). Purified PCR products were ligated into vector PMD-19T overnight (Bao Biological Engineering Co., Dalian, China). The recombinant clones were transformed into competent *Escherichia coli* DH5α cells (Tiangen, Beijing, China). 200 μL of the bacteria solution were applied to Luria-Bertani (LB) agar plates containing 100 ng/mL ampicillin, and maintained at 37°C overnight. The positive recombinant clones were selected on LB-ampicillin and confirmed by sequencing (Sangon Shanghai, China). The raw *SLC4A11* and *MFSD3* mRNA sequences were aligned using QUMA online software to analyze the degree of methylation at each CpG site. The methylation rate for each sample was calculated as the number of CpG methylated loci/the number of CpG loci.

### Statistical Analysis

One-way analysis of variance and Student’s *t*-test were used for comparisons between the control and DON treated groups. The association between an individual CG methylation and the expression levels of genes after DON-induced cytotoxicity in IPEC-J2 cells was analyzed using Pearson’s correlation analysis. All statistical analyses were performed using the SPSS 23.0 (IBM Corp., Armonk, NY, United States) or GraphPad Prism version 8.0 for Windows (GraphPad Software Inc., San Diego, CA, United States). *P* < 0.05 was considered statistically significant and all experimental samples had three replicates.

## Results

### DON-Induced Cytotoxicity in IPEC-J2 Cells

**FIGURE 1:**
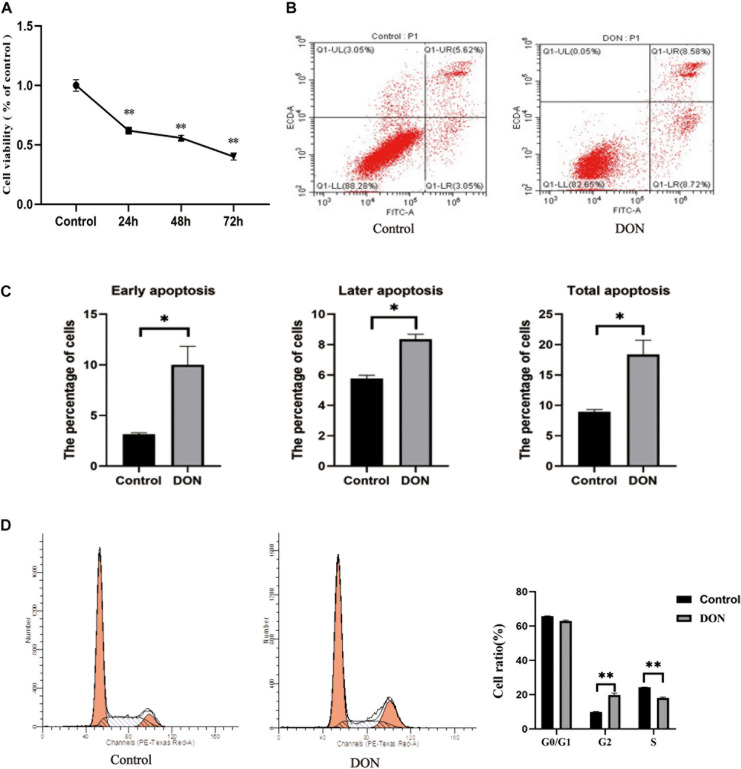
The viability, cell cycle, and apoptosis of IPEC-J2 cells in the control group and the DON treated group. **(A)** The viability of IPEC-J2 cells in the control group and the DON treated group. **(B)** Apoptosis of IPEC-J2 cells in the control group and the Deoxynivalenol (DON) treatment group was evaluated by measurement of Annexin V by flow cytometry. Apoptotic cells were Annexin V-positive and PI-negative. Q1-UL stands for nuclear debris, Q1-LL stands for living cells, Q1-LR stands for early apoptotic cells, and Q1-UR stands for later apoptotic cells **(C)** Quantification of panel **(B)**. *P < 0.05. **(D)** The cell cycle distribution in the control group and the DON treated group. **P < 0.01.

To investigate the effect of DON on the viability of IPEC-J2 cells, the cells were incubated with DON (1 μg/mL) for 24, 48, and 72 h. Cell viability was significantly lower compared with that of the control group at 24, 48, and 72 h (*P* < 0.01), indicating that DON induced increased cell death with time (Figure 1A). Flow Cytometry analysis also showed that DON increased the percentage of early, late, and total apoptotic cells significantly compared with that in the control group following 48 h of incubation (Figures 1B,C). In subsequent experiments, the cell samples were treated with DON for 48 h. Flow cytometry analysis of the cell cycle showed that after 48 h of DON treatment the cell cycle was arrested at the G2/M phase compared with the controls (*P* < 0.01), and the percentage of cells in the S phase decreased (*P* < 0.01) (Figure 1D).

### Effects of DON Induction on the Expression Levels of *SLC4A11* and *MFSD3* in IPEC-J2 Cells

**FIGURE 2:**
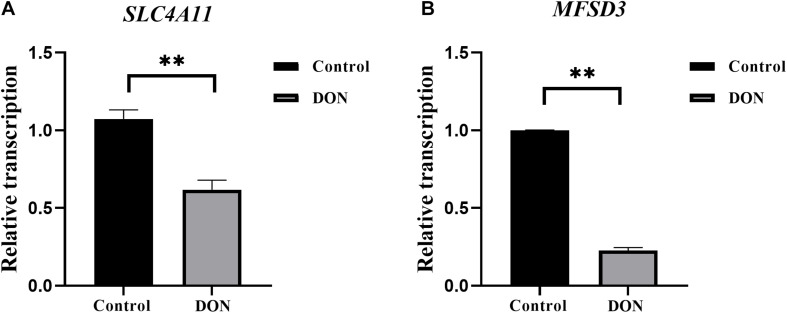
*SLC4A11* and *MFSD3* mRNA expression levels in DON-induced IPEC-J2 cells. **(A)** *SLC4A11* mRNA expression levels induced by DON. *P < 0.05. **(B)** *MFSD3* mRNA expression levels induced by DON. **P < 0.01.

The mRNA expression levels of *SLC4A11* and *MFSD3* were assessed using qRT-PCR in DON-treated IPEC-J2 cells. In the DON treated group, the mRNA expression levels of *SLC4A11* (*P* < 0.01) (Figure 2A) and *MFSD3* (*P* < 0.01) were significantly lower compared with those in the controls (Figure 2B).

### Construction of Overexpression Cell Line *SLC4A11* and *MFSD3*

**FIGURE 3:**
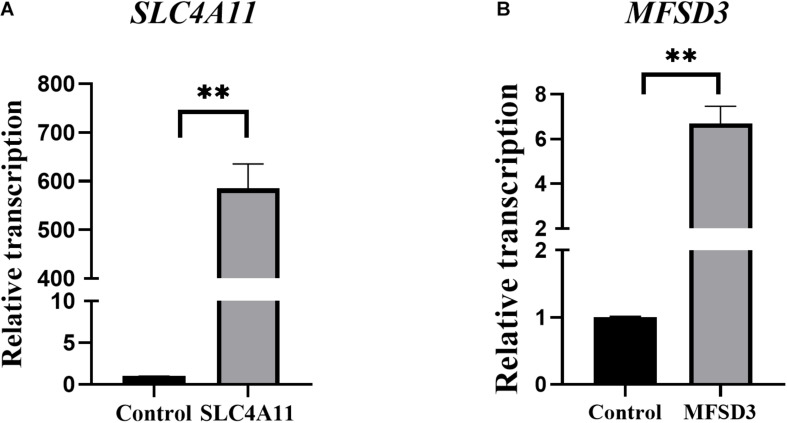
Construction of recombinant plasmids and cell lines overexpressing pEGFP-C1-SLC4A11, pEGFP-C1-MFSD3. **(A)** pEGFP-C1-SLC4A11 plasmid expression efficiency. **P < 0.01. **(B)** pEGFP-C1-MFSD3 plasmid expression efficiency. **P < 0.01.

The recombinant plasmids pEGFP-C1-SLC4A11 and pEGFP-C1-MFSD3 were verified by DNA sequencing (Supplementary Figure 1). The gene sequences of the overexpression vectors were consistent with those of the pEGFP vector, and the *SLC4A11* and *MFSD3* CDS, which indicated that plasmids were successfully constructed. After 15 days of screening with geneticin, green fluorescent cells could still be observed under the fluorescence microscope, which indicated that the recombinant fusion proteins of pEGFP-C1-SLC4A11 and pEGFP-C1-MFSD3 were stably expressed in IPEC-J2 cells (Supplementary Figure 2). The relative mRNA expression levels of *SLC4A11* in the overexpression group represent a 585.09-fold increase compared with that in the control group (*P* < 0.01) (Figure 3A). The relative expression levels of the *MFSD3* mRNA in the overexpression group representing a 6.68-fold increase compared with that in the control group (*P* < 0.01) (Figure 3B).

### Effects of Overexpression of *SLC4A11* and *MFSD3* on the Viability of IPEC-J2 Cells Induced by DON

**FIGURE 4:**
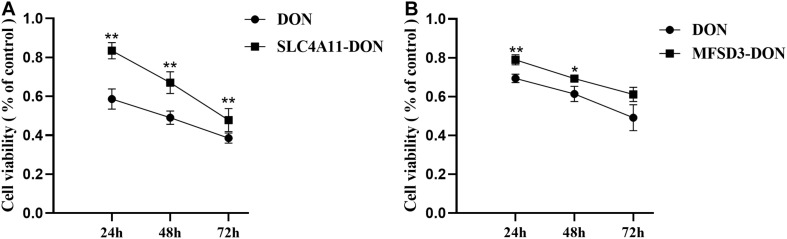
Effects of DON induction on the viability of *SLC4A11* and *MFSD3* overexpressing IPEC-J2 cell lines after 24, 48, and 72 h. **(A)** The cell viability of the *SLC4A11* cell line and DON treated group induced by DON. **P < 0.01. **(B)** The cell viability of the *MFSD3* cell line DON treated group. *P < 0.05. **P < 0.01.

*SLC4A11* and *MFSD3* overexpressing cells were incubated with DON (1 μg/mL) for 24, 48, and 72 h to detect cell viability. The results showed that the viability of the *SLC4A11* overexpression group under DON treatment was significantly increased compared to the control group at 24, 48, and 72 h (*P* < 0.01) (Figure 4A). For the *MFSD3* overexpression group under DON treatment, the viability was significantly lower that of the control group at 24 and 48 h (*P* < 0.05). There was an increasing trend at 72 h, but the changes were not significant (*P* > 0.05) (Figure 4B).

### Prediction of *SLC4A11* and *MFSD3* CpG Islands and Methylation Analysis

**FIGURE 5:**
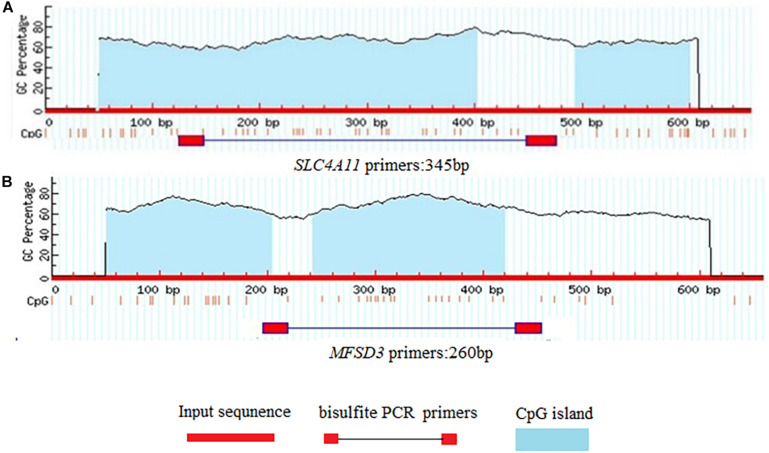
Prediction of CpG islands in gene promoter regions. **(A)** CpG islands in the *SLC4A11* gene promoter region. **(B)** CpG islands in the *MFSD3* gene promoter region.

**FIGURE 6:**
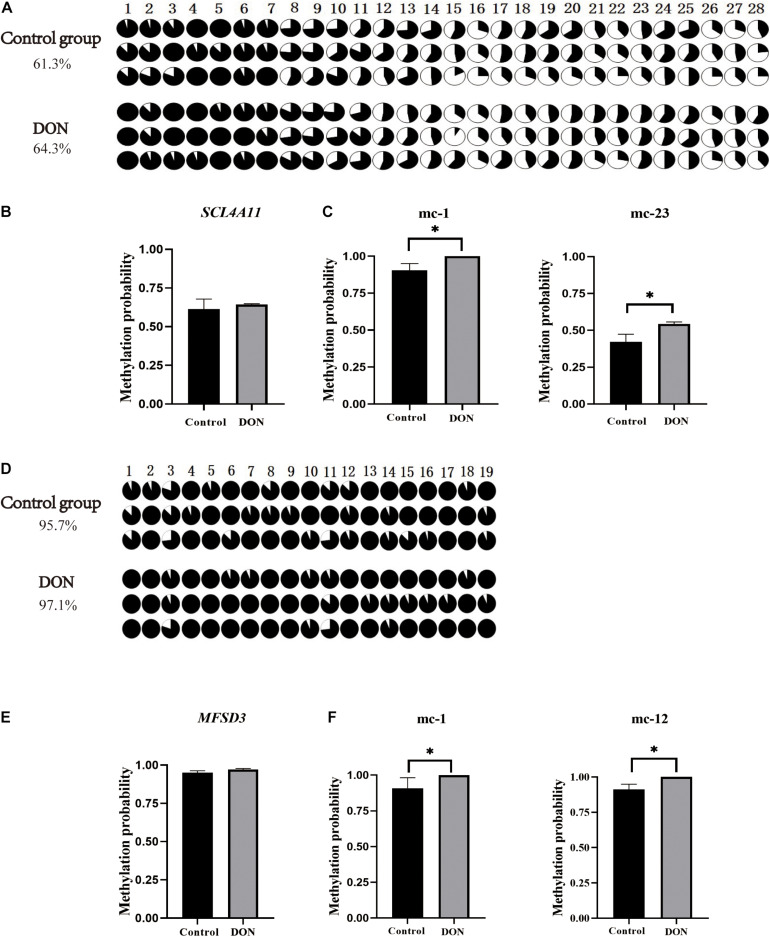
CpG island methylation analysis of the *SLC4A11* and *MFSD3* gene promoter regions. **(A)** The methylation level of CpG site of the *SLC4A11* gene. **(B)** Degree of methylation of the *SLC4A11* gene between the DON treated and control groups. **(C)** the methylation levels of the mC-1 and mC-23 sites of *SLC4A11* between the DON treated and control groups. *P < 0.05. **(D)** The methylation level of CpG site of the *MFSD3* gene. **(E)** Degree of methylation of the *MFSD3* gene between the two groups. **(F)** the methylation levels of the mC-1 and mC-12 sites of *MFSD3* between the DON treated and control group. *P < 0.05.

The CpG islands in the porcine *SLC4A11* and *MFSD3* 5′ flanking region were predicted by MethPrimer, respectively. The analysis indicated that the *SLC4A11* 5′ flanking region contained two CpG islands, and primers were designed to amplify a 345 bp fragment (Figure 5A). The *MFSD3* 5′ flanking region also contained two CpG islands, and primers were designed to amplify a 260 bp fragment (Figure 5B). After determining the CpG island regions of the porcine *SLC4A11* and *MFSD3* genes, we analyzed the rest of the gene sequences and found that the *SLC4A11* gene contained 28 CpG sites (Figure 6A) and the *MFSD3* gene contained 19 CpG sites (Figure 6D). Methylation levels of both genes in the DON group (48 h of treatment) and the control group were very high. In addition, there was no significant difference in the overall methylation degree of *SLC4A11* and *MFSD3* between the DON treated group and the control group (Figures 6B,E). Interestingly, the methylation levels of the mC-1 and mC-23 sites of *SLC4A11* and the mC-1 and mC-12 sites of *MFSD3* were significantly higher compared with those in the controls (*P* < 0.05) (Figures 6C,F).

### Correlation Between the Methylation Level of *SLC4A11* and *MFSD3* Gene Amplified Fragment and mRNA Expression

**FIGURE 7:**
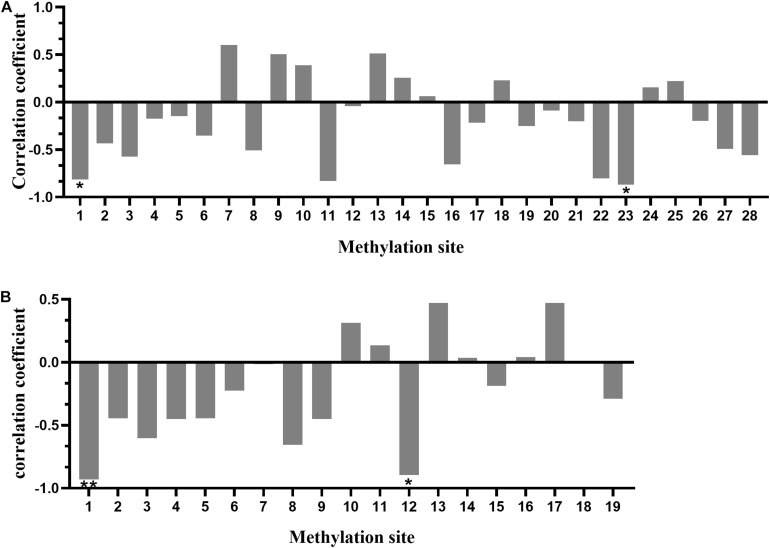
Correlation between the degree of methylation of CpG islands in the gene promoter region and mRNA expression. **(A)** Correlation between the CpG island methylation degree in the promoter region of *SLC4A11* gene and mRNA expression. *P < 0.05. **(B)** Correlation between CpG island methylation degree in the promoter region of the *MFSD3* gene and mRNA expression. *P < 0.05; **P < 0.01.

Pearson analysis was performed to examine the correlation between the methylation degree of CpG islands in the promoter regions of *SLC4A11* and *MFSD3* genes and the mRNA expression level. The methylation level of 19 (out of 28) CpG sites in *SLC4A11* CpG islands correlated negatively with the mRNA expression level, in which the methylation levels of mC-1 and mC-23 correlated significantly and negatively with the mRNA expression level (*P* < 0.05) (Figure 7A). The methylation level of 11 (out of 19) CpG sites in *MFSD3* correlated negatively with the mRNA expression level (Figure 7B), in which the methylation level of mC-1 and mC-12 correlated significantly and negatively with the mRNA expression level (*P* < 0.05).

### Prediction of Transcription Factor Binding Sites

**FIGURE 8:**
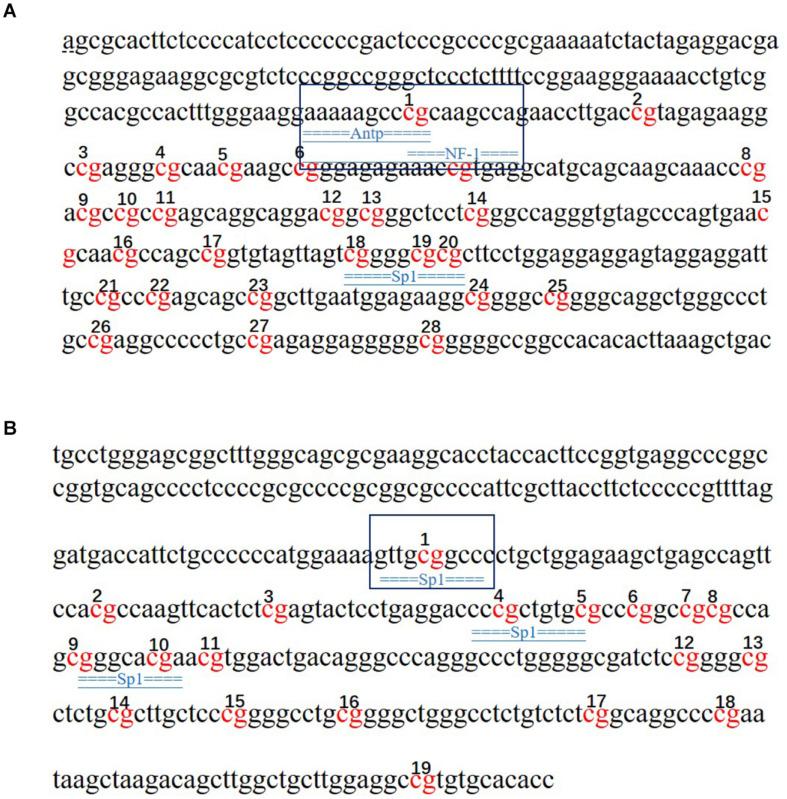
Prediction of transcription factors that bind to the *SLC4A11* and *MFSD3* genes. **(A)** Prediction of transcription factor binding sites in the *SLC4A11* gene promoter region. **(B)** Prediction of transcription factor binding sites in the *MFSD3* gene promoter region. The underlined regions represent the transcription factor binding sites, and the red bases are the CpG sites.

The potential transcription factor binding sites in the regions containing the CpG islands in the *SLC4A11* and *MFSD3* genes were predicted. The results showed that mC-1 of *SLC4A11* was located in the NF-1 and Antp binding region, while mC-23 was not located in a transcription binding site (Figure 8A). The mC-1 site of *MFSD3* was located in the Sp1 binding region, while mC-12 was not located in a transcription binding site (Figure 8B).

## Discussion

The growth and function of intestinal epithelial cells might be affected by DON through various pathological mechanisms, including the activation of cell signaling and ribosomal stress (Diesing et al., 2012). In pigs, the intestines are the main organ that absorbs DON, and most of it is absorbed in the jejunum (Diesing et al., 2011). The porcine intestinal epithelial IPEC-J2 cell line provides an ideal *in vitro* model system to study porcine-specific pathogenesis, which can show basic features similar to those *in vivo* (Goyarts and Dänicke, 2006). In this study, we examined the effects of DON on the viability, cell cycle, and apoptosis of IPEC-J2 cells. We found that DON significantly decreased cell viability, induced cell cycle arrest in the G2/M phase, and increased the apoptosis rate. Previously, it was shown that DON affects cell growth significantly by inducing IPEC-J2 cell apoptosis and arresting the cell cycle in the G2/M phase (Schierack et al., 2006), which is consistent with our results. Wang et al. (2019) showed that DON significantly increased the expression of the *BCL10* and *AEN* genes, whose protein products function in the induction and enhancement of apoptosis. Previous research also showed that DON decreases the activity of porcine endometrial cells, resulting in apoptotic phenotypes, such as mitochondrial swelling, membrane rupture, and cytoplasmic vacuolization, which hinders the synthesis of DNA, arrest the cell cycle at the G0 and G1 phases, and reduces the expression of PCNA, a key protein in the cell cycle, thus inhibiting cell proliferation (Tiemann et al., 2003; Diesing et al., 2011). Other studies have indicated the toxicological effects of DON on cell growth of different cell types via activation of the apoptosis and cell cycle arrest pathway. Our previous transcriptome analysis found that the expression levels of *SLC4A11* and *MFSD3* decreased significantly in the DON treated group. DON activates MAPKs related to differentiation and apoptosis, thereby disrupting normal cell functions. Previous research showed DON induces apoptosis and disrupts cellular homeostasis through MAPK signaling pathways in bovine mammary epithelial cells (Lee et al., 2019) and *SLC4A11* activated the MAPK pathway to stimulate cell growth and proliferation (Lopez et al., 2009). As MAPKs are important molecules in regulation of cell cycle and growth (Vithana et al., 2006), we speculate that *SLC4A11* and *MFSD3* may play a central role in DON-induced cell damage. In the present study, the expression levels of *SLC4A11* and *MFSD3* in the DON treated group were significantly lower than those in the control group. The cell viability of *SLC4A11* and *MFSD3* IPEC-J2 overexpressing cell lines under DON induction enhanced compared with the control group. *SLC4A11* and *MFSD3* can maintain nutrient physiological functions, and overexpression of *SLC4A11* and *MFSD3* may help resist the toxicity of DON. A previous study showed that relative to *SLC4A11* wild-type cells, *SLC4A11* knockout caused obvious oxidative damage to the corneal endothelial cells and depressed glutamine (Gln) catabolism, suggesting that *SLC4A11* can protect cells from Gln-induced toxicity (Bonanno and Ogando, 2018). A recent study found that *SLC4A11* plays a key role in the oxidative stress response in human corneal endothelial cells (HCEnC) mediated by the *NRF2* gene, and overexpression of *SLC4A11* in HEK 293 cells resulted in a significant increase in cell viability and reduced reactive oxygen species (ROS) in these cells compared with those in the control cells (Guha et al., 2017). In addition, DON exerts a direct toxic effect on the IPEC-J2 cells by enhancing ROS accumulation, activating NF-kB and apoptotic signaling pathways (Ruifen et al., 2019). Therefore, we hypothesized that the downregulation of *SLC4A11* and *MFSD3* expression is closely related to growth inhibition induced by DON.

DNA methylation is a significant epigenetic modification, which can regulate gene expression by promoting or inhibiting the ability of transcription factors to bind to DNA (Keshet et al., 1985; Lister et al., 2009). Liu et al. (2020) showed that DON affected the expression of growth related-genes in liver through DNA methylation. This suggested that epigenetic modifications play an important regulatory role in the mechanism of DON’s effects. In view of the role of *SLC4A11* and *MFSD3* gene expression on DON-induced injury, the present study explored the regulatory mechanism of promoter methylation on DON-induced toxicity. The results showed that the methylation levels of *SLC4A11* gene methylation sites (mC-1 and mC-23) and *MFSD3* methylation sites (mC-1 and mC-12) correlated significantly and negatively with the mRNA expression level. Previous research showed that promoter region methylation was associated with inhibition of gene expression, which occurs mainly through interference, in which the binding of transcription factors to the target gene or histone deacetylase (HDACs) is hindered (Keshet et al., 1985; Nan et al., 1998). Our study found that the mC-1 site of *SLC4A11* is located in an NF1 transcription factor binding domain and the mC-1 site of *MFSD3* is located in an Sp1 transcription factor binding domain. Sp1-like transcription regulators participate in the regulation of cell function, including cell proliferation, apoptosis, differentiation, and tumor transformation, by regulating the expression of many genes with CG-rich promoters (Black et al., 2001). NF-1 is considered as a common transcription factor (Inoue et al., 1990). However, the interaction with specific NF-1 subtypes in different cell types might contribute to the selective transcriptional activation or silencing of target genes (Chaudhry et al., 1997). ANTP belongs to Transcription factors of the homeodomain family (Bernd et al., 2008) NF-1, ANTP and Sp1 transcription factors fulfill important roles in cell proliferation and growth. Various components in different CpG islands play their respective roles in gene regulation. Some specific CpG sites have a crucial impact on the function of CpG islands and are decisive factors for the methylation of CpG islands (Mikeska et al., 2007; Barrera and Peinado, 2012). A single methylation site in the *CCR3* (C–C motif chemokine receptor 3) region is a candidate region for causing narcolepsy (Mihoko et al., 2018). Wang et al. (2020) found that changes in the methylation degree of mC-6 site would significantly change the expression of gonadotropin releasing hormone 1 (*GNRH*), indicating that the methylation level of specific sites would affect the expression level of the gene. Therefore, it can be speculated that in the regulatory regions of *SLC4A11* and *MFSD3*, hypermethylation of mC-1 in *SLC4A11* and *MFSD3* induced by DON in IPEC-J2 cells might hinder the binding of transcription factors to their target sequences, which would inhibit gene expression.

In the present study, we found that overexpression of *SLC4A11* and *MFSD3* can enhance the cell viability and alleviate the cytotoxicity of DON. The methylation levels of the mC-1 and mC-23 sites of *SLC4A11*, and the mC-1 and mC-12 sites of *MFSD3* showed significantly negative correlations with mRNA expression. These findings indicated that the *SLC4A11* and *MFSD3* genes may play important roles in regulating the growth of IPEC-J2 cells and the DON-induced cell damages. This study provided novel insights into the biological functions of *SLC4A11* and *MFSD3* genes in regulating the cytotoxic effects induced by DON, which lays important foundations for future studies on the identification of functional gene and toxic mechanisms associated with DON. Further studies are needed to use chip-assay to confirm transcription factor binding, to reveal the detailed relationship between *SLC4A11* and *MFSD3* promoter methylation and the specific mechanism of DON-induced cytotoxicity.

## Data Availability Statement

The raw data supporting the conclusions of this article will be made available by the authors, without undue reservation.

## Author Contributions

WB and SW designed the study. YX, LY, and YW acquired and interpreted the data. YX analyzed the data and was a major contributor in writing the manuscript. YX and XC prepared figures and tables. ZW, HW, and WB prepared the manuscript and supervised the study. All authors read and approved the final manuscript.

## Conflict of Interest

The authors declare that the research was conducted in the absence of any commercial or financial relationships that could be construed as a potential conflict of interest.

## Footnotes

1 https://www.ncbi.nlm.nih.gov/gene/100157069
