# Supplementary material for: SLC4A11 and MFSD3 Gene Expression Changes in Deoxynivalenol Treated IPEC-J2 Cells
Source: Front Genet. 2021 Jul 21;12:697883. doi: 10.3389/fgene.2021.697883 (PMC8335166; doi:10.3389/fgene.2021.697883)
Supplement: Supplementary file 1 [file Table_1.DOCX]

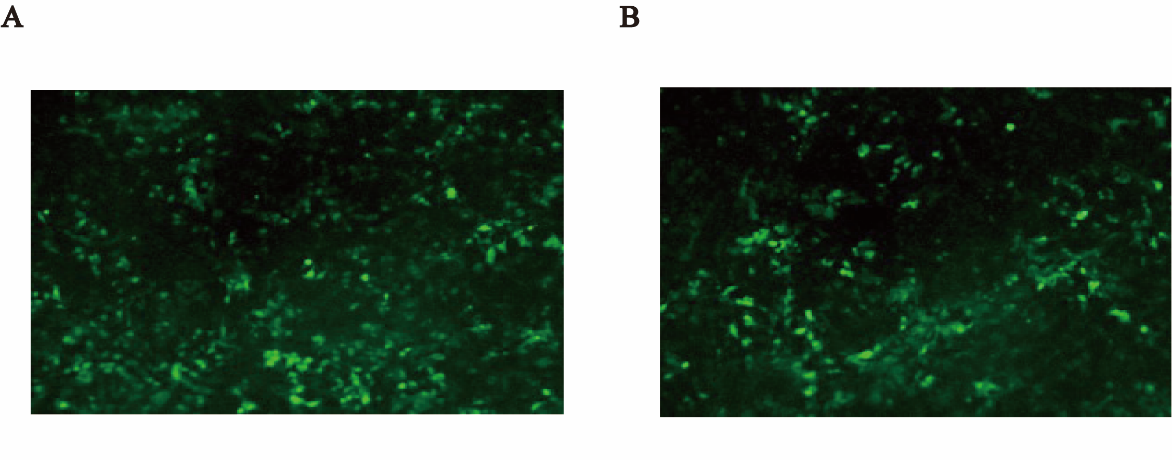

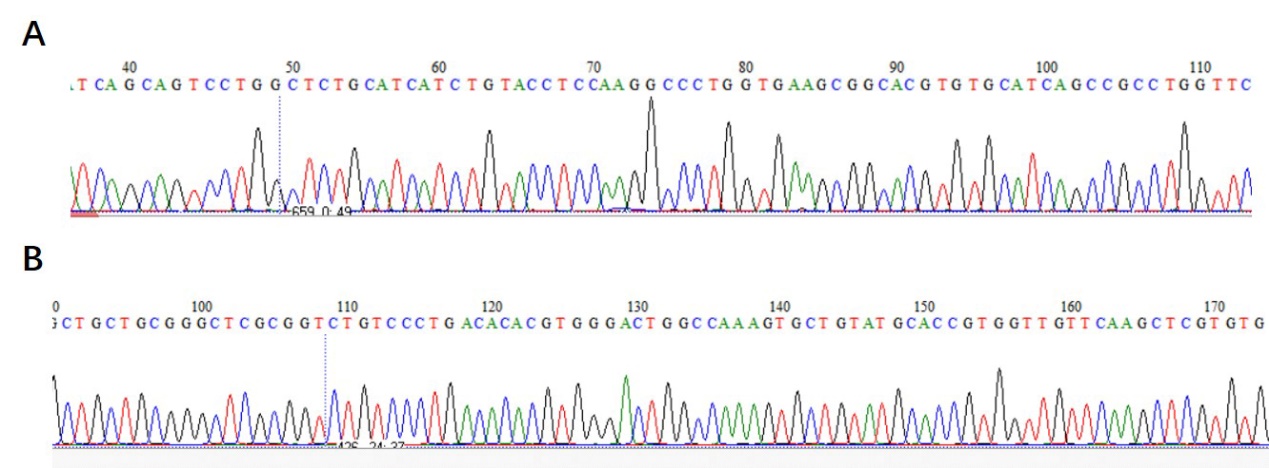
**Figure S1:** pEGFP-C1-SLC4A11 and pEGFP-C1-MFSD3 plasmid restriction enzyme digestion and sequencing results. **(A)** pEGFP-C1-SLC4A11 plasmid restriction enzyme digestion and sequencing results. **(B)** pEGFP-C1-MFSD3 plasmid restriction enzyme digestion and sequencing results.

**Figure S2:** pEGFP-C1-SLC4A11 and pEGFP-C1-MFSD3plasmid fluoresce- nce images. **(A)** pEGFP-C1-SLC4A11 plasmid fluorescence images. **(B)** pE- GFP-C1-MFSD3 plasmid fluorescence images.
